# Copper and chromium removal from industrial sludge by a biosurfactant-based washing agent and subsequent recovery by iron oxide nanoparticles

**DOI:** 10.1038/s41598-023-45729-5

**Published:** 2023-10-30

**Authors:** Tipsuda Subsanguan, Phoomipat Jungcharoen, Nichakorn Khondee, Pantita Buachan, Buddhika Prabath Abeyrathne, Nitra Nuengchamnong, Antika Pranudta, Suttipong Wannapaiboon, Ekawan Luepromchai

**Affiliations:** 1 Center of Excellence in Microbial Technology for Marine Pollution Treatment (MiTMaPT), Department of Microbiology, Faculty of Science, Chulalongkorn University, Bangkok, Thailand; 2 Department of Environmental Engineering, Faculty of Engineering, Khon Kaen University, Khon Kaen, Thailand; 3 Department of Natural Resources and Environment, Faculty of Agriculture Natural Resources and Environment, Naresuan University, Phitsanulok, Thailand; 4 International Program in Hazardous Substance and Environmental Management (IP-HSM), Graduate School, Chulalongkorn University, Bangkok, Thailand; 5 Science Laboratory Centre, Faculty of Science, Naresuan University, Phitsanulok, Thailand; 6 Synchrotron Light Research Institute, Nakhon Ratchasima, Thailand

**Keywords:** Biotechnology, Environmental sciences, Materials science

## Abstract

Industrial wastewater treatment generates sludge with high concentrations of metals and coagulants, which can cause environmental problems. This study developed a sequential sludge washing and metal recovery process for industrial sludge containing > 4500 mg/kg Cu and > 5000 mg/kg Cr. The washing agent was formulated by mixing glycolipid, lipopeptide, and phospholipid biosurfactants from *Weissella cibaria* PN3 and *Brevibacterium casei* NK8 with a chelating agent, ethylenediaminetetraacetic acid (EDTA). These biosurfactants contained various functional groups for capturing metals. The optimized formulation by the central composite design had low surface tension and contained relatively small micelles. Comparable Cu and Cr removal efficiencies of 37.8% and 38.4%, respectively, were obtained after washing the sludge by shaking with a sonication process at a 1:4 solid-to-liquid ratio. The zeta potential analysis indicated the bonding of metal ions on the surface of biosurfactant micelles. When 100 g/L iron oxide nanoparticles were applied to the washing agent without pH adjustment, 83% Cu and 100% Cr were recovered. In addition, X-ray diffraction and X-ray absorption spectroscopy of the nanoparticles showed the oxidation of nanoparticles, the reduction of Cr(V) to the less toxic Cr(III), and the absorption of Cu. The recovered metals could be further recycled, which will be beneficial for the circular economy.

## Introduction

Metal-containing sludges are produced from both municipal and industrial wastewater treatment plants. For municipal sludge, the removal of metals will enable the land application of sludge for the improvement of soil quality and promote plant growth^1^. However, industrial wastewater and sludge have high concentrations of metals; thus, they should be used as a secondary resource of metals^2^. Industrial wastewater is usually treated by chemical coagulants such as aluminum sulfate (alum), sodium aluminate, ferric sulfate, and lime (CaO), which lead to large quantities of potentially toxic industrial sludge produced after coagulation^3^. When compared to soil and municipal wastewater sludge, industrial sludge is more difficult to wash due to the presence of coagulants and higher metal concentrations. It is thus necessary to develop economically viable and sustainable approaches for recovering and recycling metals from industrial sludge^4^. This study focused on copper (Cu) and chromium (Cr) removal from industrial sludge because they have been reported in various industries. For example, wastewater from the chemical, distillery, pulp and paper, tannery, textile, and petroleum industries contains Cu and Cr as major heavy metals^2^.

Chemical washing agents such as surfactants, chelating agents, inorganic acids, and organic acids have been used for the removal of metals from industrial sludge^5,6^. The washing procedure is simple, cost-effective, and fast; however, the washing agent should be environmentally friendly, biodegradable, and inexpensive to prevent secondary environmental pollution^6^. In this study, biosurfactant-based washing agents were formulated by mixing biosurfactants from 2 bacteria, *Weissella cibaria* PN3 and *Brevibacterium casei* NK8, which have different molecular structures and properties^7,8^. Each biosurfactant has been investigated for its potential application in environmental remediation and agricultural enhancement^8,9^, however, the mixing of these two biosurfactants has never been conducted. Biosurfactants are emerging as suitable alternatives to petroleum-derived surfactants because they are more sustainable in many stages of the product lifecycle^10^. In addition, biosurfactants are complex molecules with various functional groups and are usually composed of carbohydrates, amino acids, lipids, and fatty acids^11^. Yang et al.^6^ suggested that heavy metal removal is attributed to the washing agent’s functional groups (e.g., hydroxyl, carboxyl, carbonyl) because they can capture metal ions with metal-binding groups in sludge via ion exchange, chelation, and electrostatic adsorption. In fact, a mixture of rhamnolipid and saponin has been found to have high metal removal efficiency from municipal wastewater sludge due to the presence of various functional groups, such as hydroxyl, carboxyl, and amine groups^12^.

The interactions between biosurfactants and various metals are influenced by the concentrations of acids or alkalines, biomolecules, the charge of the metal, and soil properties^13^. Cu and Cr have different charges, so they might interact with biosurfactant molecules in different ways. This study, therefore, optimized the biosurfactant-based washing agent by varying the concentrations of each biosurfactant (i.e., PN3 and NK8 biosurfactants) and pH values. The synergistic effect of mixed surfactants is due to diverse self-assembly dynamics, which can promote micellar aggregation and lead to the shortest wetting time, smallest contact angle, and lowest surface tension^14,15^. In soil, an appropriate mixing of several surfactants can inhibit the adsorption of individual surfactants onto soil and each other, which decreases the loss of sorbed surfactants and increases their pollutant washing efficiency^16,17^. Consequently, the optimized biosurfactant-based washing agent was expected to interact well with both Cu and Cr ions but had low sorption capacity with industrial sludge.

This study further used ethylenediaminetetraacetic acid (EDTA) as a chelating agent and organic builder in the washing agent formulation. The combined use of surfactants and chelating agents is often adopted to enhance the mobilization of metallic contaminants^16^, while organic builders such as EDTA, nitrilotriacetic acid (NTA), disodium 3-oxopentanedioate (ODA), iminodisuccinic acid (IDA), and sodium citrate (Na-C) can be used in conjunction with surfactants to remove Ca^2+^ and Mg^2+^ ions and thus to lower the content of surfactants in the washing formulations^18^. In addition, sludge washing was carried out under shaking and ultrasonication conditions. The ultrasonication technique creates cavitation in the sample solution and can be used to facilitate the mass transfer of the metal ions to the extraction agent^19^. Ultrasonication has been applied to increase the soluble percentages of Cr, Cu, Ni, and Zn in sewage sludge^20^ but not in industrial sludge.

After the washing process, Cu and Cr were recovered from the washing agent by iron oxide nanoparticles. Magnetic iron oxide nanoparticles (Fe_3_O_4_) have been developed and applied for the removal of heavy metals from aqueous solutions because of their high surface area, superparamagnetic characteristics, and reactivity^21,22^. In addition, they can be readily isolated from the solutions using an external magnetic field. The surface of magnetite nanoparticles can be modified with various organic ligands, such as humic acid, polymer, and fatty acids, to stabilize nanoparticles and improve their ability to remove metal contaminants, including Cr(VI) and Cu(II)^23–29^. However, this study used bare magnetite nanoparticles to avoid the complicated synthesis process. It was expected that the residual biosurfactants in the washing agent would promote the activity and stability of nanoparticles. There are reports on the benefits of biosurfactant molecules on magnetite nanoparticles such as preventing agglomeration and oxidation, improving chemical resistance, and increasing the availability of pollutants during nanoparticle applications^30,31^.

In summary, this is the first study to develop a simple process for washing industrial sludge and subsequently recovering Cu and Cr for further recycling. An industrial sludge sample was collected from a waste treatment company in Thailand and used as a model. The sludge contained high concentrations of Cu (> 4500 mg/kg) and Cr (> 5000 mg/kg) and had been produced in large amounts annually. Biosurfactants from *Weissella cibaria* PN3 and *Brevibacterium casei* NK8 were selected based on their different molecular structures and potential cost reduction when using food and agro-industrial wastes as substrates^7,8^. The nontoxic and recyclable Fe_3_O_4_ nanoparticles were synthesized by a simple coprecipitation method, and the effect of residual biosurfactant and pH in the washing agent on the activity of nanoparticles was investigated. The acquired process for sludge treatment and metal recovery will be beneficial for both environmental management and the circular economy.

## Materials and methods

### Industrial sludge

An industrial sludge sample was collected in one batch (~ 100 kg) from different points of the sludge drying unit at the conventional industrial wastewater treatment plant, General Environment Conservation Public Company Limited (GENCO), Thailand. The wastewater was mostly generated by chemical companies such as metal plating factories, steel factories, and dyeing factories. The samples had high moisture content, so they were dried for 7 days at ambient temperature (28–30 °C). The samples with high moisture content were dried for 7 days at ambient temperature (28–30 °C). Dried sludge samples from different collection points were mixed until homogeneous and sieved to less than 2 mm particle size before use in the experiment (Supplementary Fig. 1a). The characteristics of the industrial wastewater sludge, including organic carbon (OC), total nitrogen (TN), total phosphorus (TP), total potassium (TK), and pH, were analyzed at the Soil Plant and Agricultural Material Testing and Research Unit, Central Laboratory and Greenhouse Complex, Kasetsart University, Kamphaeng Saen. The concentrations of heavy metals (Cu, Cr, Ni, Mn, and Zn) were analyzed at the Environmental Research Institute of Chulalongkorn University (ERIC).

### Bacteria, biosurfactants, and chemicals

Biosurfactants, including glycolipids, lipopeptides, and phospholipids, were produced by *Weissella cibaria* PN3 and *Brevibacterium casei* NK8. Glycolipids were produced by *Weissella cibaria* PN3 with 2% (v/v) soybean oil as a substrate as described in Subsanguan et al.^7^. This bacterium produced glycolipids in the form of extracellular and cell-bound molecules. Cell-bound glycolipid from *Weissella cibaria* PN3 has been applied as a main component in biosurfactant-based dispersants for marine oil spill remediation^9^; thus, this study only focused on the use of extracellular glycolipid. *Brevibacterium casei* NK8 produces a mixture of lipopeptide and phospholipids^8^. The bacterium was cultured under alkaline conditions to allow the utilization of 5% (w/w) coconut oil cake as a substrate following Khondee et al.^8^. Crude PN3 and NK8 biosurfactants were extracted from cell-free broth containing biosurfactant molecules using solvents according to Subsanguan et al.^7^ and Khondee et al.^8^, respectively. The stock solution of biosurfactant was prepared by dissolving crude biosurfactant in phosphate buffer solution (PBS, pH 8.0) to the required concentration. The surface tension and CMC value of PN3 biosurfactant were 31.3 mN/m and 1.6 g/L, respectively, whereas NK8 biosurfactant had a surface tension of 27.2 mN/m and a CMC value of 1.1 g/L. All other chemicals were of analytical grade and purchased from Sigma-Aldrich Co., LLC.

### Formulation of a biosurfactant-based washing agent

Biosurfactant-based washing agents were initially formulated by preparing biosurfactant solution at the concentration three times its critical micelle concentration (3 × CMC), e.g., 4.8 g/L PN3 biosurfactant and 3.3 g/L NK8 biosurfactant. At this concentration, PN3 biosurfactant forms stable micelles^9^, which could promote the capture of metal ions. The biosurfactant solution was later mixed with 1 M EDTA, and the pH was adjusted with 2 M NaOH or 37% HCl to the desired values. For the biosurfactant mixture, PN3 and NK8 biosurfactant solutions were mixed at a 1:1 mass ratio before adding EDTA and adjusting the pH. The prepared biosurfactant-based washing agents were vortexed until homogeneous and kept at room temperature (Supplementary Fig. 2). The control solution was 1 M EDTA at pH 7. To optimize the concentration of each biosurfactant and pH of the formulation, central composite design (CCD)-response surface methodology (RSM) was applied. The independent variables (factors) were PN3 and NK8 biosurfactant concentrations and pH, which varied in the range of 2–6 g/L and pH 3–5, respectively. The dependent variables (responses) were the removal efficiency of Cu and Cr. The accuracy of this model using STATISTICA10 program software (StatSoft Tulsa, OK, USA) was shown by the observation that the experimentally determined actual values closely corresponded to the predicted values provided by the CCD-RSM design. The high Cu and Cr removal efficiency demonstrated the optimum combination of process variables. Optimization design responses were evaluated using analysis of variance (ANOVA). The interaction effects and significance of the variables influencing the concentrations of biosurfactants were investigated using ANOVA. The consequent values for the *R*^2^ coefficient of determination and the *R*^2^ adjusted value were used to evaluate the polynomial. The model significance corresponds to the probability “*p*” function. Under optimal conditions, the obtained model's validity was experimentally verified in triplicate.

### Optimization of the washing process

The washing process was carried out with a 1:4 solid-to-liquid ratio. Briefly, 10 g of industrial wastewater sludge and 40 mL of washing solution were placed in a 125 mL Erlenmeyer flask. For mechanical soil washing, all flasks were shaken on a rotary shaker at 200 rpm for 10 min at room temperature. The combined soil washing (shaking with ultrasonication) was conducted by placing the flasks in a sonicator bath for 10 min of ultrasonication at 37 kHz and 80 W and then transferring them to a rotary shaker for mechanical soil washing for another 10 min according to Park and Son^32^. The washing solution was separated from the solid by centrifugation at 4 °C and 8000 rpm for 10 min and filtered with a 0.45 µm sterile filter to collect the washing agent for metal analysis. Then, the washed sludge (solid part) was rinsed with deionized water (DI) using the same washing process method.

To increase the efficiency of Cu and Cr removal, the washing process was repeated for 3 cycles. After the washing and rinsing process in the 1st cycle, the washed sludge was supplemented with a fresh biosurfactant-based washing agent to begin the 2nd cycle and processed with a similar procedure until finishing the 3rd cycle. The washed sludge and all solutions, including the washing agent and rinsing water, from each cycle were collected at the end of the experiment to measure the concentrations of Cu and Cr. In addition, the washing agent before and after use was analyzed for surface tension, zeta potential, particle size, and pH to determine the mechanisms relevant to metal removal. The accumulated removal efficiency was determined by combining the Cu and Cr removal efficiency from each cycle.

### Synthesis and application of iron oxide nanoparticles for Cu and Cr recovery from the washing agent

Magnetite iron oxide nanoparticles were synthesized based on the simple and effective coprecipitation of ferric and ferrous salts and adapted as reported by Jungcharoen et al.^33,34^. For one batch of synthesis, 100 mL of 0.5 M HCl containing 2.16 M FeCl_2_ and 4.32 M FeCl_3_ (1:2 molar ratio) was added to 0.5 M NaOH (250 mL) with continuous stirring at room temperature. A black precipitate of Fe_3_O_4_ was immediately obtained, and the pH of the suspension was adjusted to 8.5 by using NaOH. Then, the suspension was washed twice with ultrapure water at pH 8.5. The washed suspension (250 mL) contained approximately 200 g/L Fe_3_O_4_, which could be diluted to a desired Fe_3_O_4_ concentration before use.

The efficiency of Fe_3_O_4_ on Cu and Cr recovery was initially tested with synthetic water containing 250 mg/L Cu(II) (from Cu(NO_3_)_2_·3H_2_O) and 150 mg/L Cr(VI) (from K_2_Cr_2_O_7_) with and without biosurfactant to determine the effect of biosurfactant residues on Fe_3_O_4_. Synthetic water with biosurfactants (+ GP) was prepared with 5 g/L each of PN3 and NK8 biosurfactants and 1 M EDTA to simulate the F3 formulation. To recover both Cu and Cr, the study used a high concentration of Fe_3_O_4_ (100 g/L) and varied the pH at 5.5 and 8. The pH of the washing agent (waste F3) was approximately 8.0 (Table 3). However, Cu(II) removal is usually carried out at pH 5.5 to 6^35–37^, while Cr(VI) is effectively removed at pH 2 to 4^38^. It should be noted that although working at low pH increases the removal efficiency for Cr(VI), it also favors the leaching of iron by magnetite nanoparticles^39^. Consequently, pH 5.5 was selected for comparison. The washing agent was filtered through 0.22 µm cellulose acetate membranes to remove suspended matter prior to the experiment. Equilibrium studies of Cu(II) and Cr(VI) sorption on Fe_3_O_4_ nanoparticles were carried out at 200 rpm for a sufficient period of time (24 h) following (Rajput et al.^19^; Simeonidis et al.^39^). The magnetite nanoparticles were separated from the liquid solution by magnetic decantation and analyzed by X-ray absorption spectroscopy. The remaining solution was collected and filtered through a 0.22 µm filter. The residual Cu and Cr concentrations in the liquid solution were determined by inductively coupled plasma atomic emission spectrophotometry (ICP‒OES).

### Analysis

#### Characterization of biosurfactants and washing agents

The surface tension (ST) of crude biosurfactant dissolved in phosphate buffer solution was measured using a digital tensiometer (Kruss, K10ST, Germany) at 25 °C using the plate method. The critical micelle concentration (CMC) was determined from a plot of surface tension versus biosurfactant concentration. Liquid chromatography coupled with electrospray ionization quadrupole time-of-flight mass spectrometry (LC–ESI–QTOF–MS/MS) was performed to analyze the identity of the purified biosurfactants from *Weissella cibaria* PN3 using an Agilent 6540 Q–TOF–MS spectrometer (Agilent Technologies Inc.) coupled with an Agilent 1260 Infinity Series high-performance liquid chromatography system (Agilent Technologies Inc.) with processes and conditions according to Khondee et al.^8^. The zeta potential and particle (micelle) size of the washing agent before and after use were determined by electrophoretic and dynamic light scattering methods using a Zetasizer Nano ZS (Malvern Panalytical Ltd) by adding the sample in a cuvette at particular pH conditions and room temperature. To identify the participant functional groups of the biosurfactants, the washing agents before and after use were dried at 40 °C and analyzed by Fourier transform infrared (FTIR) spectroscopy (Spectrum GX, PerkinElmer) in wavenumbers ranging from 4000 to 400 cm^−1^ at a resolution of 0.3 cm^−1^.

#### Characterization of nanoparticles

The particle size of bare magnetite nanoparticles was determined by transmission electron microscopy (TEM; TECNAI, G220) operated at 200 kV. For sample preparation, the nanoparticles were dispersed in ethyl alcohol for 30 min in a sonicator. The sample was then dropped on standard holy carbon films and dried at room temperature. It was transferred to a Cu grid (EMS, USA) for analysis under the microscope. The number of nanoparticles (approximately 100 particles) was measured by ImageJ software. Powder X-ray diffraction (XRD) patterns of the prepared materials were measured in Debye–Scherrer geometry at ambient temperature at Beamline 1.1W (Multiple X-ray Techniques) at the Synchrotron Light Research Institute, Thailand, using monochromatic synchrotron X-ray radiation of 12 keV (wavelength of 1.0332 Å) for all samples. The powder sample was packed in a Kapton capillary with a diameter of 0.3 mm and then aligned using a goniometer head. During the measurement, the capillary was constantly rotated. The diffraction pattern was recorded by a 1-dimensional strip detector (Mythen6K 450, DECTRIS^®^) in the 2θ range of 10°–70°.

For further characterizations of chemical speciation and local structure of metals on nanoparticles, HA520E-Fe was investigated by applying X-ray absorption spectroscopy (XAS) at the BL8–XAS of Synchrotron Light Research Institute (SLRI), Nakhon Ratchasima, Thailand. The X-ray absorption near edge structure (XANES) spectra were collected at the Fe-*K* edge (7112 eV) and Cr-*K* edge (5989 eV) using energy scanning by a double crystal monochromator (DMC) equipped with a Ge (220) crystal. For Fe K-edge absorption, both Fe standards and samples were measured in transmission mode. For Cr K-edge adsorption, the Cr standard was measured in transmission mode, while all samples were obtained in fluorescent mode due to the trace concentration of the element. X-ray absorption near edge structure (XANES) results of the Cu K-edge were further performed at Beamline 1.1 W (Multiple X-ray Techniques). The photon energy was calibrated using the Cu foil standard for the zero-oxidation state at 8979 eV. The sample measurements were conducted at ambient temperature and pressure in fluorescence mode using a 19-element Ge detector (Canberra) to detect the signal after absorption by the samples. The standard materials representing Cu foil, CuO and Cu_2_O, were measured in transmission mode. The scanning photon energy is used with an energy step of 0.3 eV for XANES spectra and 0.05k in k-space for EXAFS spectra with a collection time of 7 s per point. Moreover, XAS data processing was performed using ATHENA software.

#### Metal analysis

All heavy metals (Cu, Cr, Ni, Mn, and Zn) were analyzed by the Environmental Research Institute of Chulalongkorn University (ERIC). Sludge samples were digested before measuring heavy metal concentration by USEPA 3015 Microwave Assisted Acid Digestion, whereas all sample solutions were not applied. The dilution of liquid sample (i.e., digested sample and washing solution) was carried out in 2 steps of 30 times and 10 times. The concentrations of heavy metals in diluted solution were measured by an inductively coupled plasma atomic emission spectrophotometer (ICP‒OES). Cu and Cr spiked samples were used to determine the recovery efficiency, which was in the range of 100 ± 15%.

The percentages of Cu and Cr removal efficiency were calculated from their concentrations in the industrial sludge before (*S*_*0*_) and after washing (*S*_*t*_), as shown in Eq. (1).

1 $$\% \;{\text{Removal }} = \left[ {\frac{(S0 - St)}{{S0}}} \right] \times 100$$

The percentages of Cu and Cr recovery efficiency were calculated from their concentrations in the washing agent before (*C*_0_) and after treatment (*C*_*t*_), as shown in Eq. (2).

2 $$\% \;{\text{Recovery}} = \left[ {\frac{(C0 - Ct)}{{C0}}} \right] \times 100$$

## Results and discussion

### Characteristics of industrial sludge

The industrial sludge from a local waste treatment company contained high concentrations of Cr (5153.7 ± 87.0 mg/kg) and Cu (4691.0 ± 50.1 mg/kg) and low concentrations of Ni, Mn and Zn at less than 500 mg/kg (Table 1). The concentrations of Cr and Cu were much higher than those in other wastewater sludges. For example, sludge samples from wastewater treatment plants in China that treat both industrial and domestic wastewater have average Cr and Cu values of 212.5 and 1261.1 mg/kg, respectively, as reported by Tang et al.^12^, and 377.6 and 695.8 mg/kg, respectively, as reported by Guo et al.^40^. Sludge from a petrochemical company in Qatar contains 2.14% of total metal with iron as the major metal, while Cr and Cu are present in low concentrations at 33.3 and 22.8 mg/kg, respectively^41^. Nonetheless, Cr can be found at a very high concentration (279,400 mg/kg) along with Ni but not Cu in electroplating sludge^42^. Since Cr and Cu are the main metals in sludge from various wastewater treatment plants, they must be removed and recovered before sludge disposal.

**Table 1:** Characteristics of the industrial wastewater sludge before and after washing by biosurfactant-based washing formulations.

Parameters	Initial sludge	Sludge after washing	
		F3	F4
Cr (mg/kg)	5153.70 ± 87.02	2801.67 ± 51.01	3155.67 ± 22.19
Cu (mg/kg)	4691.00 ± 50.12	2516.67 ± 36.12	2214.00 ± 15.39
Ni (mg/kg)	435.67 ± 14.47	418.33 ± 3.68	417.67 ± 9.61
Mn (mg/kg)	312.67 ± 4.17	303.33 ± 2.49	308.33 ± 5.51
Zn (mg/kg)	209.00 ± 5.00	204.33 ± 1.53	207.33 ± 5.86
OC (g/kg)	6.00 ± 0.10	32.70 ± 0.19	36.40 ± 0.15
TN (g/kg)	0.70 ± 0.01	0.90 ± 0.01	0.70 ± 0.01
TP (g/kg)	2.00 ± 0.01	5.40 ± 0.01	5.50 ± 0.03
TK (g/kg)	0.10 ± 0.01	0.04 ± 0.01	0.06 ± 0.01
pH	8.6 ± 0.15	7.5 ± 0.11	8.0 ± 0.10

*OC* organic carbon, *TN* total nitrogen, *TP* total phosphorus, *TK* total potassium, *F3* formulation containing 5.0 g/L PN3 biosurfactant and 5.0 g/L NK8 biosurfactant, *F4* formulation containing 4.5 g/L PN3 biosurfactant and 5.5 g/L NK8 biosurfactant.

The industrial sludge had low organic carbon, TN, TP and TK contents (Table 1), which corresponded with the coagulation process in the treatment plant. The characteristics of this sludge were different from those of municipal sludge, where the organic carbon, TN, TP and TK contents were almost 10 times higher than those in this study and mainly originated from biomass in the treatment plant. For example, the concentrations of TOC, TN, TP and TK in sludge from a study by Tang et al.^12^ are 291.95 g/kg, 14.20 g/kg, 23.79 g/kg, and 5.83 g/kg, respectively. Municipal sludge primarily absorbs heavy metals from wastewater via passive sorption and active uptake of biomass^1^. Thus, the metal speciation in municipal and industrial sludge would be different and would affect the efficiency of the metal removal method. It is therefore necessary to develop a specific metal removal method for industrial sludge.

### Efficiency of individual and mixed biosurfactants for Cu and Cr removal from industrial sludge

**Figure 1:**
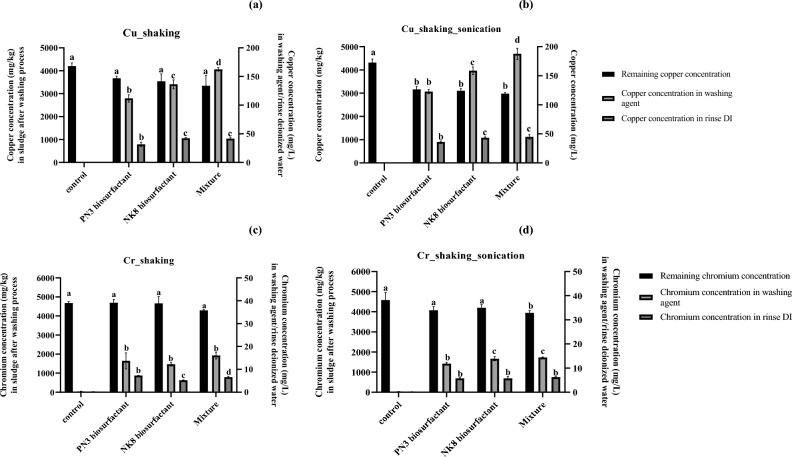
Cu and Cr concentrations in sludge (mg/kg), washing agent (mg/L) and rinse deionized water (mg/L) after washing the industrial sludge with single and mixed biosurfactants at pH 7.0. The washing conditions included shaking (**a**,**c**) and shaking with sonication (**a**,**d**). The initial copper and chromium concentrations in sludge were 4691 and 5154 mg/kg, respectively. The control solution was 1 M EDTA at pH 7.

When the industrial sludge was washed with biosurfactants from *Weissella cibaria* PN3 and *Brevibacterium casei* NK8 as single or mixed solutions, Cu was removed from the sludge more than Cr in all treatment conditions (Fig. 1). The concentrations of Cu in the washing agent and rinsing water ranged from 111.7–162.3 mg/L and 31.1–42.2 mg/L, respectively, while the Cr concentrations in the washing agent and rinsing water ranged from 12.2–16.1 mg/L and 5.2–7.3 mg/L, respectively. This was due to the strong binding of Cr ions to sludge. The metal removal efficiency of the control solution was very low, with only 0.1 mg/L Cu and 0.06 mg/L Cr in the washing solution and 0.06 mg/L Cu and 0.23 mg/L Cr in the rinsing water (Fig. 1). The results suggested that biosurfactant molecules along with EDTA were responsible for metal desorption from sludge and solubilization into the washing agent. The remaining concentrations of Cu in sludge after washing with all biosurfactant solutions were significantly different from those after washing with the control solution when the combined washing process of 10 min shaking and 10 min sonication was used (Fig. 1). Similarly, this washing condition led to significant Cr removal when mixed biosurfactants were used. The application of ultrasonication for increasing metal solubilization from sewage sludge has been reported (Yesil et al.^20^); however, this is the first study to combine sonication with a biosurfactant-based washing agent for industrial sludge treatment. The process was conducted in a closed system, so the potential environmental impacts of EDTA would be minimized. Similarly, Gluhar et al.^43^ demonstrated the advantage of EDTA over other biodegradable chelators for soil washing with ReSoil technology where the process water is reused in a closed loop and EDTA is recycled.

When comparing each biosurfactant, the Cu and Cr removal efficiencies of the NK8 biosurfactant were higher than those of the PN3 biosurfactant, as seen from the higher Cu and Cr concentrations in the washing agents, especially from the combined washing process (Fig. 1b,d). This was probably due to the presence of various functional groups in the NK8 biosurfactant, which is composed of zwitterionic lipopeptides and phospholipids^8^. The mixing of NK8 and PN3 biosurfactants increased the Cu removal efficiency but not Cr, as seen from the higher concentrations of Cu in the mixed biosurfactant than in the single biosurfactant (Fig. 1a,b). On the other hand, Tang et al.^12^ reported that a mixture of rhamnolipid and saponin with increasing mass ratios removed Cr, Mn, and Ni from sludge at higher efficiencies than Cu, Zn, and Pb. The different findings could be due to the different types of biosurfactants used in this study. To remove both Cu and Cr from the industrial sludge, the biosurfactant mixture was optimized by varying the pH and mass of each biosurfactant in further experiments.

### Optimization of biosurfactant-based washing agents

**Figure 2:**
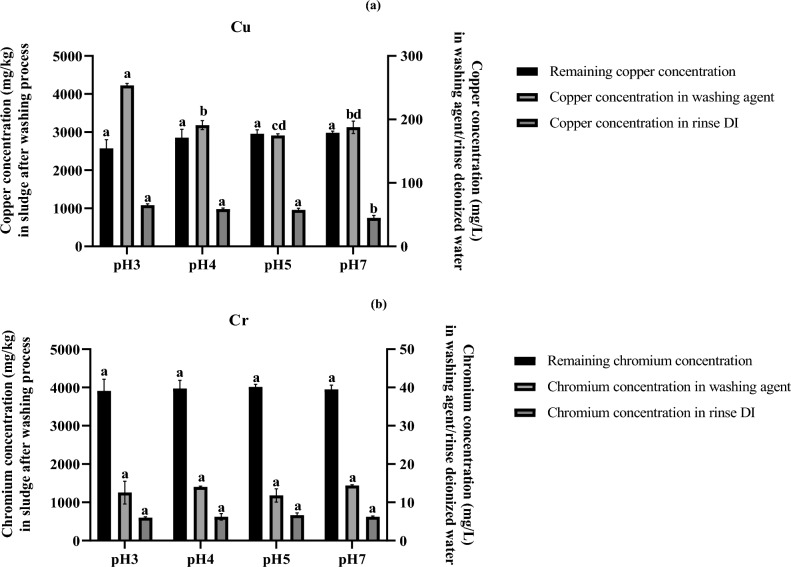
Concentrations of Cu (**a**) and Cr (**b**) in sludge (mg/kg), washing agent (mg/L) and rinse deionized water (mg/L) after washing with a mixture of PN3 biosurfactant (4.8 g/L) and NK8 biosurfactant (3.3 g/L) at different pH values.

Low-pH liquid is usually used to extract metals from sludge, and pH is the major parameter affecting metal speciation, sorption and mobilization as well as the complexation of metal-chelating agents^1,4^. From section “Efficiency of individual and mixed biosurfactants for Cu and Cr removal from industrial sludge”, the biosurfactant-based washing agents should contain 4.8 g/L PN3 biosurfactant, 3.3 g/L NK3 biosurfactant and 1 M EDTA, thus the mixture was initially optimized by lowering the pH from 7 to 3. This study did not investigate pH values below 3 to avoid potential harmful health effects of biosurfactant-based washing agents. The maximum Cu removal efficiency was found from the biosurfactant mixture at pH 3, where the concentration of Cu in the washing agent was significantly higher than those from other pH values (Fig. 2). However, lowering the pH did not enhance the removal of Cr, as seen from the similar Cr concentrations in sludge, washing agent, and rinsing water at all pH values (Fig. 2). Cr in industrial effluent exists in the form of anionic ions, and at pH values between 2.0 and 6.0, Cr exists in Cr_2_O_7_^2−^ and HCrO_4_^−^^44^, while Cu is usually in the form of cationic ions.

The washing condition was shaking with sonication. The initial copper and chromium concentrations in the industrial sludge were 4691 and 5154 mg/kg, respectively. Since many factors could affect the metal removal efficiency, the biosurfactant-based washing agents were further optimized by central composite design and response surface plots using 3 factors, 1 block and 16 experimental runs (Supplementary Table 1). When the model was plotted with varying masses of PN3 and NK8 biosurfactants and fixing the pH at 3.0, 4.0, and 5.0, the highest removal efficiencies of either Cu or Cr were achieved (Supplementary Fig. 3). The best formulation for Cu removal, F1, had a pH of 3.8 and contained PN3 and NK8 biosurfactants at 2.7 g/L and 6.9 g/L, respectively. From the prediction, this formulation could remove 46.2% Cu but had low efficiency for Cr removal (8.4%) (Table 2). Similarly, F2 at pH 4.1 and containing 6.6 g/L PN3 and 4.9 g/L NK8 biosurfactants could remove Cr (41.0%) but had low Cu efficiency (24.8%) (Table 2). These predicted values correlated well with the experimental values (Table 2). To select the formulations for simultaneously removing Cu and Cr, polynomial equations were obtained by regression coefficient analysis as stated in Eqs. (3) and (4) for Cu and Cr removal efficiencies, respectively.

3 $$Z = - 58.85 + 22.03*x - 1.88*x^{2} + 17.03*y - 0.98*y^{2} - 1.28*x*y - 0.84*3.*x - 0.08*3.*y + 19.12$$

4 $$Z = - 113.79 + 12.83*x - 1.20*x^{2} + 11.70*y - 1.75*y^{2} + 0.80*x*y - 0.19*4.*x + 0.01*4.*y + 86.06$$

where Cu and Cr removal efficiency is the response (z) and x and y are the concentrations of PN3 biosurfactant and NK8 biosurfactant, respectively.

**Table 2:** Removal efficiencies of copper and chromium from industrial sludge after washing the industrial sludge with biosurfactant mixtures and formulations obtained from the response surface plot.

Formulations	PN3 biosurfactant (g/L)	NK8 biosurfactant (g/L)	pH	Removal efficiency (%)			
				Predicted values		Experiment values	
				Cu	Cr	Cu	Cr
Mixture 1	4.8	3.3	3.0	–	–	45.12 ± 4.86	24.11 ± 5.91
Mixture 2	4.8	3.3	4.0	–	–	39.23 ± 4.75	22.89 ± 4.08
Mixture 3	4.8	3.3	5.0	–	–	36.99 ± 2.21	22.11 ± 1.31
Mixture 4	4.8	3.3	7.0	–	–	36.48 ± 1.05	23.38 ± 2.18
F1	2.7	6.9	3.8	46.21	8.42	44.69 ± 4.47	9.16 ± 1.29
F2	6.6	4.9	4.1	24.83	41.00	22.56 ± 5.04	38.81 ± 1.37
F3	5.0	5.0	4.0	38.01	37.52	37.75 ± 1.47	38.41 ± 2.59
F4	4.5	5.5	4.0	41.16	33.69	40.61 ± 3.14	33.59 ± 3.40

Two formulations, F3 and F4, at pH 4.0 were obtained from the equations for further studies. From the experiment, F3 contained 5.0 g/L each of PN3 and NK8 biosurfactants, which led to comparable Cu and Cr removal efficiencies of 37.8% and 38.4%, respectively (Table 2). F4 removed 40.6% Cu and 33.6% Cr and contained 4.5 g/L PN3 biosurfactant and 5.5 g/L NK8 biosurfactant (Table 2). Both formulations contained 1 M EDTA. Lu et al.^44^ reported that Cr can interact with a cationic surfactant, CTAB, through electrostatic forces and can later be removed from aqueous solution by foam fractionation made from a binary mixture of CTAB and saponin. The molecules of NK8 biosurfactant contain both cationic and anionic groups^8^, while the molecules of PN3 biosurfactants contain anionic groups from glycolipid structures (Subsanguan et al.^7^). The results confirmed that both biosurfactants were necessary to capture Cu and Cr from industrial sludge and that the pH of the biosurfactant-based washing agents was another important parameter affecting Cu and Cr removal efficiency.

### Application of the optimized biosurfactant-based washing agents

**Figure 3:**
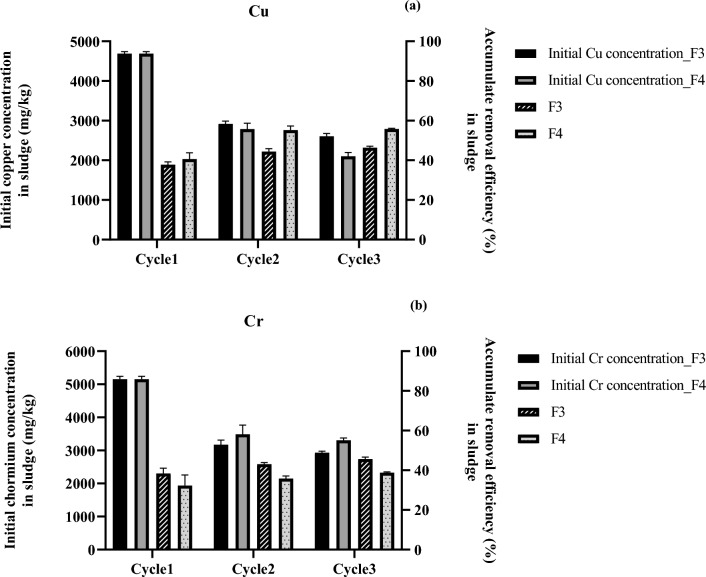
Concentrations and accumulated removal efficiency of copper (**a**) and chromium (**b**) after rewashing the industrial sludge by biosurfactant-based washing agents (F3 and F4) for 3 cycles.

The characteristics of the sludge after washing with formulations F3 and F4 are shown in Table 1. The concentrations of Ni, Mn, and Zn changed slightly from the beginning, which was probably due to the specific removal efficiency of the formulation. However, the increasing concentrations of OC, TN, and TP in the washed sludge suggested the sorption of some biosurfactant molecules on sludge particles. To increase the Cu and Cr removal efficiency, the optimized biosurfactant-based washing agents, F3 and F4, were later applied to wash the industrial sludge for 3 cycles. The residual Cu and Cr in the washed sludge, washing agent, and rinsing water after repeated washing cycles are shown in Supplementary Fig. 4 and Supplementary Table 2. The results indicated that the biosurfactant-based washing agents captured Cu and Cr to a higher extent than rinsing water in all washing cycles. In addition, Cu and Cr were mainly removed from the sludge in the 1st washing cycle, and the accumulated removal efficiencies for both metals were not much increased in the 2nd and 3rd washing cycles (Fig. 3). When compared between formulations at the 3rd washing cycle, F3 had comparable accumulated removal efficiencies for Cu (46.3%) and Cr (45.6%), while F4 removed more Cu (55.8%) than Cr (38.8%).

The residual Cu and Cr were probably bound tightly to the sludge particles and chemical coagulants used for wastewater treatment; thus, the application of repeated washing cycles could not increase the removal efficiency. Similarly, 5% saponin solutions at pH 2 removed only 24.2% of 8041 mg/kg Cr from tannery sludge after multiple washes because the chromium in tannery sludge tightly bound to colloidal particles and organic matter^45^. The removal of metals from industrial sludge is usually more difficult than that from municipal sludge and contaminated soil due to the higher concentrations and the presence of organic compounds and other coagulants. Consequently, the repeated washing cycles were not appropriate for industrial sludge, and the washed industrial sludge after the 1st cycle should be processed by other techniques to remove the remaining metals. Nonetheless, the application of multiple washing steps might be applied to sludge with lower metal concentrations. For example, Tang et al.^12^ found that a combined rhamnolipid and saponin had increased metal binding intensities, and the Cu (1261 mg/kg), Zn (682 mg/kg), Cr (213 mg/kg), Pb (68 mg/kg), Ni (126 mg/kg), and Mn (923 mg/kg) extraction efficiencies were 62%, 74%, 60%, 15%, 68%, and 64% after the 3rd washing of sludge from a combined industrial and domestic wastewater treatment plant, respectively.

Washing agent type and concentration, solution pH, solid-to-liquid ratio (S/L) and washing time are major factors contributing to heavy metal removal efficiency^6^. The washing conditions obtained from this study were a 1:4 solid-to-liquid ratio, 10 min shaking, and 10 min sonication. Raiput et al.^19^ also showed that ultrasonication offers great potential for practical applications. They used ultrasonication-assisted extraction for the removal of lead metal ions from aqueous systems using imidazolium-based ionic liquids as the sole extracting agent followed by the application of salting-out agents to reduce the release of ionic liquid cations in the aqueous phase. The solid-to-liquid ratio (1:4) used in this study was higher than that in other studies, which usually conducted sludge washing at 1:12.5^45^, 1:50^12^, and 1:80^6^. This washing process with high solid‒liquid ratio used low volume of the washing agent, which was probably due to the efficiency of both biosurfactant-based washing agents and the combined shaking and sonicating washing conditions. This condition is desirable for a scale-up washing process. Although the study focused on industrial sludge, the obtained biosurfactant-based washing agent has a high potential for metal removal from municipal sludge. It is expected that municipal sludge will require lower amounts of biosurfactant-based washing agent due to the lower metal content. The developed sludge washing process is considered as a green alternative because it consumes low energy and low amount of washing agent and the biosurfactants-based washing agents are bio-based, weakly acid, and solvent-free formulations.

### Characteristics of biosurfactant-based washing agents before and after use

The physiochemical characterization of biosurfactant-metal systems was conducted by performing surface tension, particle size, zeta potential, and FTIR with the washing agents before and after use. The used F3 had the lowest increase in surface tension (22%), lowest increase in particle size (17%), and highest increase in zeta potential (43%) (Table 3). These characteristics corresponded with its excellent performance of heavy metals detachment and solubilization, as seen from the high Cu and Cr removal efficiency (Table 2). The biosurfactant produced by *Weissella cibaria* PN3 is a glycolipid biosurfactant due to its high sugar and lipid contents^7^. LC–ESI–QTOF–MS/MS was used to confirm its characteristics, which obtained fragmented ions of amines and fatty amides as hydrophilic moieties and fragmented ions of sphinganines and fatty acids as hydrophilic moieties (Supplementary Table 3; Supplementary Fig. 5). Consequently, the biosurfactant produced from *Weissella cibaria* PN3 could be identified as a complex glycolipid (glycosphingolipid and glycopeptidolipid). Therefore, the PN3 biosurfactant contained a high density of negatively charged ions due to the presence of hydroxyl (–OH) and carboxylate (–COO) ions. NK8 biosurfactants are zwitterionic biosurfactants that contain both cations and anions^8^. The presence of various ions from mixed zwitterionic–anionic biosurfactants could reduce the intermolecular electrostatic repulsions, and in turn, monomers (molecules) form closely packed structures of micelles, resulting in lower surface tension and smaller micelle size. Ma et al.^46^ reported that the variations in the CMC, surface compositions, micelle compositions and structure of zwitterionic–anionic surfactant mixtures are interpreted as a strong attractive synergistic interaction that is asymmetric with composition. The solubilization and mobilization of heavy metals could occur when surface tension and micelle size were sufficiently lowered by mixed anionic PN3 biosurfactants and zwitterionic NK8 biosurfactants.

**Table 3:** Characteristics of biosurfactant-based washing agents and biosurfactant solutions before and after use for industrial sludge washing.

Formulations*	Before use			After use					
	Surface tension (mN/m)	Zeta potential (mV)	Particle size (nm)	pH	Surface tension (mN/m)	Zeta potential (mV)	Particle size (nm)	Cu** (mg/L)	Cr** (mg/L)
Mixture pH 3	35.5 ± 0.5	− 9.9 ± 0.7	321.3 ± 6.0	8.4	58.2 ± 1.5	− 6.6 ± 0.1	443.1 ± 7.2	253.3 ± 3.3	12.6 ± 3.0
Mixture pH 4	36.0 ± 0.6	− 14.7 ± 0.5	413.1 ± 4.4	8.2	56.0 ± 1.6	− 11.5 ± 0.2	588.5 ± 8.7	190.9 ± 7.1	14.0 ± 0.2
Mixture pH 5	36.0 ± 0.3	− 16.0 ± 0.4	645.4 ± 7.3	8.4	57.0 ± 1.0	− 10.1 ± 0.4	687.2 ± 9.3	174.5 ± 2.7	11.8 ± 1.7
Mixture pH 7	35.0 ± 0.4	− 17.1 ± 0.4	816.3 ± 5.6	8.2	49.4 ± 0.6	− 10.9 ± 0.2	960.5 ± 8.6	187.7 ± 9.8	14.4 ± 0.2
F1 (pH 3.8)	36.0 ± 1.0	− 12.7 ± 0.3	306.4 ± 4.5	8.6	52.4 ± 0.9	− 7.4 ± 0.5	836.2 ± 9.4	291.3 ± 4.6	12.4 ± 1.1
F2 (pH 4.1)	36.0 ± 0.5	− 12.4 ± 0.1	418.3 ± 6.3	8.2	46.2 ± 1.4	− 7.5 ± 0.4	954.0 ± 7.8	115.6 ± 5.5	146.9 ± 1.71
F3 (pH 4.0)	35.0 ± 0.4	− 12.7 ± 0.1	405.3 ± 3.8	7.5	45.1 ± 0.4	− 7.2 ± 0.1	487.8 ± 5.0	275.0 ± 6.2	144.1 ± 10.3
F4 (pH 4.0)	36.5 ± 0.7	− 12.5 ± 0.3	321.6 ± 3.3	8.0	48.4 ± 1.3	− 7.4 ± 0.3	721.0 ± 4.2	283.0 ± 4.6	113.2 ± 9.7
PN3 (pH 4)	33.5 ± 1.2	− 17.1 ± 1.3	656.7 ± 2.2	8.3	44.2 ± 0.8	− 9.8 ± 0.5	917.8 ± 7.8	111.7 ± 6.4	13.7 ± 3.5
NK8 (pH 4)	37.3 ± 0.5	− 12.0 ± 0.8	525.7 ± 7.7	8.5	57.6 ± 1.8	− 5.1 ± 0.6	810.5 ± 5.6	136.3 ± 7.8	12.2 ± 0.8

*The compositions of mixture and formulations (F1–F4) are in Table 2. PN3 and NK8 were biosurfactant solutions at 5 g/L (3.1 × CMC PN3 biosurfactant and 5.6 × CMC NK8 biosurfactant).

**The concentrations of Cu and Cr in the washing agent are also shown in Figs. 1 and 2.

One of the mechanisms of heavy metal removal by biosurfactants is electrostatic and van der Waals forces, which occur between the surface-active layer and the ions in sludge^47^. The zeta potential of the washing solution was investigated both before and after use to understand the interactions between biosurfactant micelles and heavy metals (Table 3). All washing formulations showed a lower negative charge density after sludge washing, demonstrating that heavy metals were bonded with the surface of micelles. The interaction of positive ions of heavy metals with negative ions of biosurfactants resulted in an increase in the zeta potential.

Functional groups that bind to metal ions could be identified by FTIR analysis. The obtained spectra of biosurfactant-based washing agents (F3 and F4) before and after use were measured by an FTIR spectrometer (Supplementary Fig. 6). The main functional groups of F3 and F4 before use were –OH (3340–3351 cm^−1^), –CN (1582 cm^−1^), –COOH (1406–1407 cm^−1^), –CO (1078–1079 cm^−1^), and –CH (519–523 cm^−1^). The FTIR peaks could shift in position or change in intensity when comparing the spectrum changes of each agent before and after washing, showing that there was interaction between the functional groups of agents and metal ions in the sludge. The peaks of F3 and F4 after use were shifted to 3379–3381, 1591, 1351–1404 and 1108–1110 cm^−1^, indicating the bond strength of hydroxyl, amine or amide, carboxyl, and carbonyl groups participating in the soil washing process by ion exchange or complexation with heavy metals^48–50^. The peaks at 519 and 523 cm^−1^ disappeared, while new peaks at 607, 615 and 711 cm^−1^ appeared. The occurrence of intense bands at 590–780 cm^−1^ is due to aromatic C–H vibrations, which can provide π-electrons for binding to metal ions and forming metals containing heterocyclic complexes^51–53^.

### Recovery of Cu(II) and Cr(VI) from the washing agent by Fe_3_O_4_ nanoparticles

**Figure 4:**
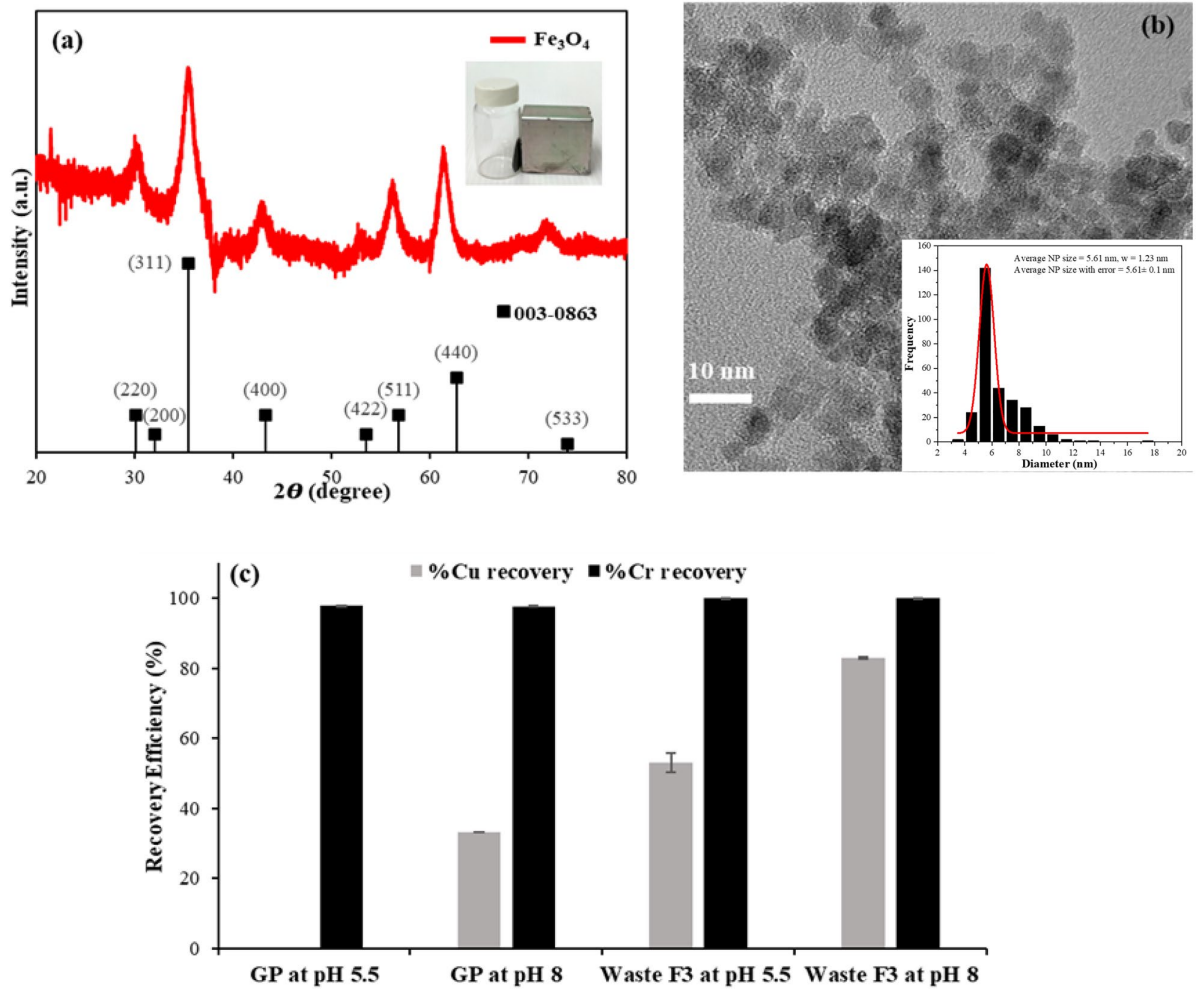
Characterization of the prepared magnetite Fe_3_O_4_ nanoparticles by powder X-ray diffraction (XRD) (**a**) and transmission electron microscopy (TEM) with a particle size distribution graph in the inset (**b**). The efficiency of Fe_3_O_4_ nanoparticles on Cu and Cr recovery was investigated with the synthetic solution containing biosurfactants and EDTA (GP) and the washing agent (Waste F3) (**c**).

The powder X-ray diffraction (XRD) pattern of Fe_3_O_4_ revealed that iron oxide nanoparticles were crystalline in nature and represented the cubic structure of Fe_3_O_4_ (magnetite), as shown in Fig. 4a. Such results are consistent with standard XRD data of Fe_3_O_4_ (JCPDS No. 003-0863)^54^. The characteristic diffraction peaks with 2θ at 30.15°, 35.37°, 43.31°, 53.69°, and 62.77° were attributed to the crystal planes of Fe_3_O_4_ (220), (311), (400), (422), (511), and (440), respectively. The high degree of peak broadening corresponded to the nanosized particles of Fe_3_O_4_. Hence, Fe_3_O_4_ nanoparticles were successfully synthesized. The TEM image showed that the nanoparticles synthesized by the coprecipitation method were nearly spherical with an average particle size of 5.61 ± 0.1 nm (Fig. 4b). The small particles tend to aggregate because of the magnetic forces between these particles^54^.

The pH of the medium is a key factor in the adsorption process because it has an impact on both the active sites of the adsorbent and the metal chemistry in solution^35,55–57^. Figure 4c shows the application of magnetite nanoparticles in synthetic water (GP) and the washing agent (waste F3) used for Cu and Cr recovery. The initial pH values of the tested solutions were 5.5 and 8, while the concentrations of Cu and Cr in synthetic water (GP) were 250 mg/L and 150 mg/L and in waste F3 were 275 mg/L and 144 mg/L, respectively. The forms of Cu and Cr in both liquid samples were Cu(II) and Cr(VI), respectively (Supplementary Fig. 7). The recovery of Cu(II) increased with increasing pH for both synthetic water (0 to 33%) and waste F3 (53 to 83%) because acidic pH conditions lead to an abundance of hydronium ions (H_3_O^+^) in the solution, which causes competition between hydronium ions and Cu(II) (Cu^2+^, Cu(OH)^+^ at lower pH) for adsorption onto nanoparticles^35,36,58,59^.

The Cr recovery process by Fe_3_O_4_ was more efficient than that of Cu, which was due to the heterogeneous redox process of Cr(VI). Fe_3_O_4_ nanoparticles have been reported as a powerful material for Cr(VI) removal^26,27^. However, the recovery of Cr from both was quite the same from pH 5.5 to 8, which was due to the high concentration of Fe_3_O_4_ nanoparticles (100 g/L) and long contact time of the 24-h experiment. Similarly, Simeonidis et al.^39^ found that it is possible to completely remove 100 µg/L Cr(VI) from contaminated wastewater by increasing the contact time to 5 h with 1 g/L Fe_3_O_4_ at pH 7.5. Hence, the best conditions for the recovery of Cu(II) and Cr(VI) from the wastewater were at pH 8, which was the pH of the washing agent after use. The results indicated that the metal recovery process could be carried out after the sludge washing process without any pH adjustment.

**Figure 5:**
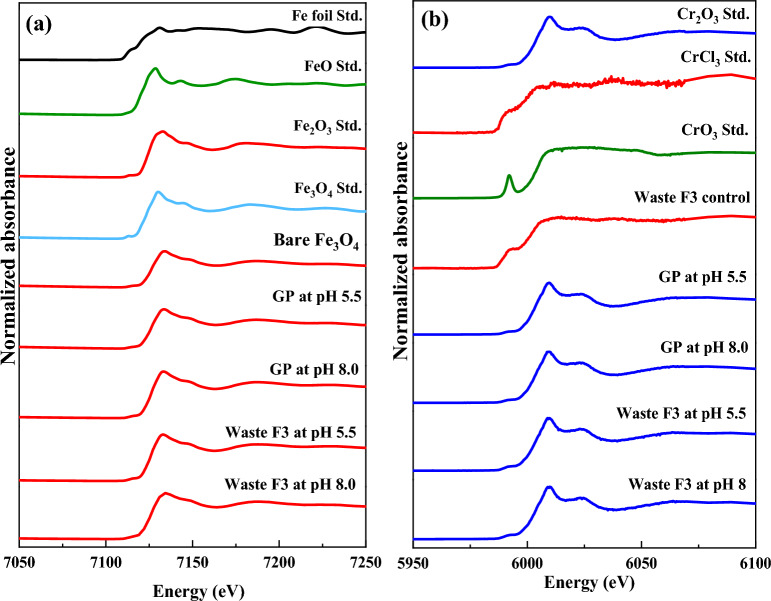
Normalized XANES spectra of Fe_3_O_4_ nanoparticles after reacting with metals in the synthetic solution containing biosurfactants and EDTA (GP at pH 5.5 and 8.0) and the washing agent (Waste F3 at pH 5.5 and 8.0) at the Fe *K*-edge (**a**) and Cr *K*-edge (**b**). The reference spectra of standard chemicals (Fe foil, FeO, Fe_2_O_3_, Cr_2_O_3_, CrCl_3_, and CrO_3_), bare Fe_3_O_4_ nanoparticles, and waste F3 control (before the recovery experiment) are shown for comparison.

The chemical speciation and local structure of metals on nanoparticles were investigated by Fe, Cr, and Cu K-edge XANES analysis. This XANES technique is very sensitive to the local electronic configuration of the photoadsorbing atom^37,60,61^. As shown in Fig. 5a, the Fe K-edge XANES spectra of the samples under different conditions were compared with those of standard reference materials with various oxidation states and chemical speciation, namely, Fe foil (Fe(0)), FeO, Fe_2_O_3_, and Fe_3_O_4_. Herein, we observed the similarity of the absorption edge energy of all samples at 7127 eV and XANES fingerprint spectra as that of the Fe_2_O_3_ standard, which agrees well with previous reports^62,63^. For Cr(VI) *K*-edge XANES spectra, Cr(III) was detected on the surfaces of nanoparticles, as seen from the similar fingerprint spectra with the Cr_2_O_3_ standard (Fig. 5b). Consequently, the oxidation of the surface of Fe_3_O_4_ nanoparticles was observed to be Fe_2_O_3_, while Cr(VI) was reduced to Cr(III) and then absorbed at the Fe-based nanoparticles. This observation indicated (i) the oxidation of Fe_3_O_4_ nanoparticles in the presence of chromium ions due to the redox potential and (ii) the high sensitivity toward the oxidation reaction of the bare Fe_3_O_4_ nanoparticles from oxygen in the ambient air. Cr(VI) reduction in the presence of Fe_3_O_4_ nanoparticles occurred for all concentrations of Fe_3_O_4_, as shown in Supplementary Fig. 7a. In addition, the Cu K-edge XANES spectra showed the adsorption of Cu(II) by Fe_3_O_4_ nanoparticles under all tested conditions (Supplementary Fig. 7b).

In this study, the efficiency of Cu recovery from synthetic water was lower than that from the washing agent (Fig. 4c). This was probably due to the higher concentration of biosurfactants in synthetic water, which could disturb the interaction between the metal and nanoparticles. During the washing process, some biosurfactants were sorbed by sludge particles; thus, the washing agent contained residual biosurfactant molecules, as seen from the increase in surface tension (Table 3). The effect of biosurfactant concentrations on magnetite nanoparticles should be further investigated. Nonetheless, this study found low precipitation of nanoparticles in all conditions. It was possible that the biosurfactant molecules could attach to the surfaces of Fe_3_O_4,_ which made them less aggregated. The larger surface area of nanoparticles would allow them to undergo many successive reduction and oxidation cycles during the recovery process. The nanoparticles were easily separated from the washing agent by a permanent magnet. After that, Cu and Cr can be desorbed from these nanoparticles by using acids such as 0.1 M HCl^35^ or 0.01 M HNO_3_^55^ and later used as raw materials. This process also regenerates the nanoparticles. In general, Fe_3_O_4_ nanoparticles maintain nearly 80–85% of their initial adsorption capacity after four to five adsorption/desorption cycles without any mass loss^35,55^. Since the characteristics of the washing agent were different from those of other wastewaters, it is necessary to further investigate the reusability and treatability of this wastewater after separating the magnetite nanoparticles. Overall, the synthesized magnetite nanoadsorbent has high potential for application in metal recovery-based industrial processes. The mass production of Fe_3_O_4_ nanoparticles can be carried out because the synthesis process is simple and uses only non-expensive and non-toxic reagents.

## Conclusions

This study develops a novel sequential industrial sludge washing and metal recovery process, which is simple and environmentally friendly. Cu and Cr in the industrial sludge were efficiently removed by the F3 formulation, which contained biosurfactants from *Weissella cibaria* PN3 and *Brevibacterium casei* NK8 and a chelating agent, EDTA. The synergistic effect of these biosurfactants and EDTA at pH 4.0 allowed the mobilization of both Cu and Cr ions from sludge to the biosurfactant aggregates. Then, Cu and Cr were recovered from the washing agent by employing magnetite nanoparticles (Fe_3_O_4_). X-ray diffraction and X-ray absorption spectroscopy of the nanoparticles showed the reduction of Cr(V) to the less toxic Cr(III), the absorption of Cu on nanoparticles, and the oxidation of nanoparticles. In summary, the sequential washing and recovery process can be used to prevent metal contamination from sludge disposal, reduce the cost of sludge treatment, and finally promote a circular economy from the utilization of industrial sludge as a resource for metal extraction.

### Supplementary Information

Supplementary Information.

## Data Availability

All data generated or analysed during this study are included in this published article [and its supplementary information files].
