# Supplementary material for: Copper and chromium removal from industrial sludge by a biosurfactant-based washing agent and subsequent recovery by iron oxide nanoparticles
Source: Sci Rep. 2023 Oct 30;13:18603. doi: 10.1038/s41598-023-45729-5 (PMC10616064; doi:10.1038/s41598-023-45729-5)
Supplement: Supplementary file 1 — Supplementary Information. [file 41598_2023_45729_MOESM1_ESM.docx]

**Supplementary Materials**

**Copper and chromium removal from industrial sludge by a biosurfactant-based washing agent and subsequent recovery by** **iron oxide nanoparticles**

**(A)**

**
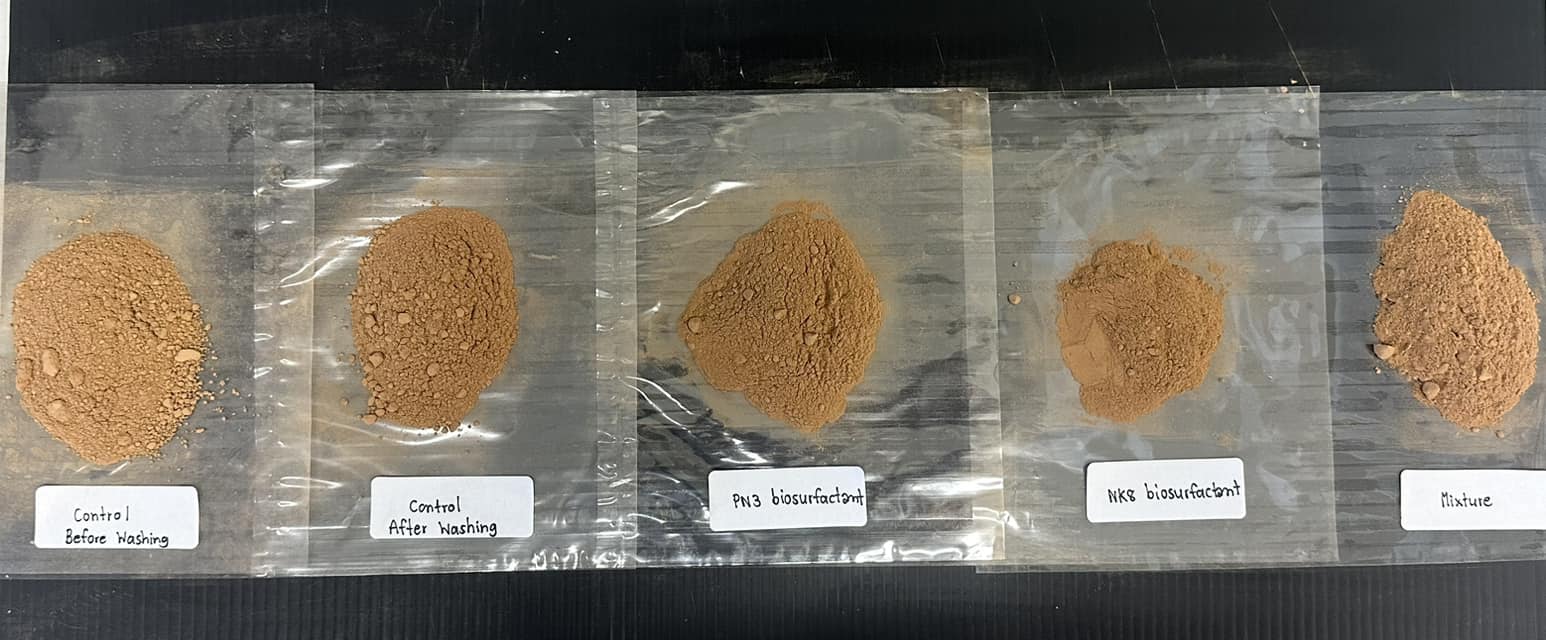
**

**DI water**

**PN3**

**NK8**

**Mixture**

**Original sludge**

**(B)**


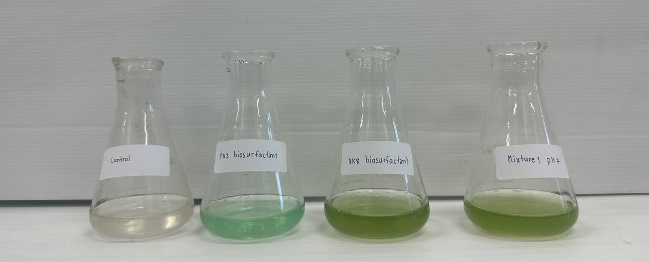


**DI water**

**PN3**

**NK8**

**Mixture**

**Supplementary Figure 1.** Industrial sludge samples before and after the washing process (A) and used washing agents (B). The washing agents were DI water, PN3 biosurfactant, NK8 biosurfactant, and mixed biosurfactant which were investigated in Section 3.2.

**
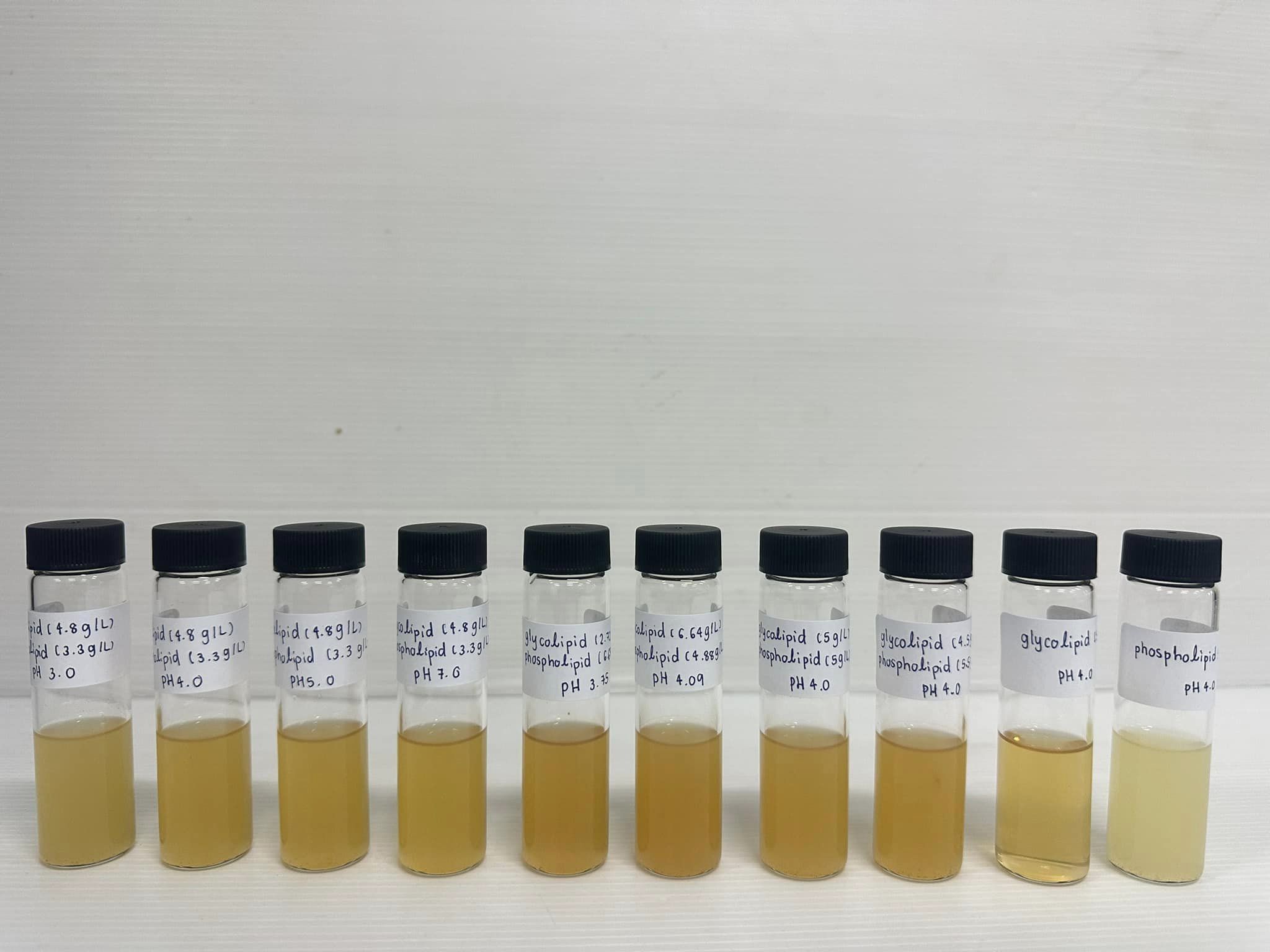
**

**Supplementary Figure 2.** Biosurfactant-based washing agents with varying concentrations of PN3 and NK8 biosurfactants and pH values.

**Supplementary Table 1.** Central composite design (CCD) of 3 factors including concentrations of PN3 and NK8 biosurfactants and pH for formulating biosurfactant-based washing agents for removing Cu (A) and Cr (B) from the industrial sludge.

**(A) Cu removal efficiency**

| Run no. | PN3 biosurfactant (g/L) | NK8 biosurfactant (g/L) | pH | Removal efficiency of Cu (%) |
| --- | --- | --- | --- | --- |
| 1 | 2.00 | 2.00 | 3.00 | 20.86 |
| 2 | 2.00 | 2.00 | 5.00 | 19.67 |
| 3 | 2.00 | 6.00 | 3.00 | 45.96 |
| 4 | 2.00 | 6.00 | 5.00 | 49.93 |
| 5 | 6.00 | 2.00 | 3.00 | 23.51 |
| 6 | 6.00 | 2.00 | 5.00 | 21.31 |
| 7 | 6.00 | 6.00 | 3.00 | 34.00 |
| 8 | 6.00 | 6.00 | 5.00 | 25.58 |
| 9 | 0.64 | 4.00 | 4.00 | 15.80 |
| 10 | 7.36 | 4.00 | 4.00 | 13.21 |
| 11 | 4.00 | 0.64 | 4.00 | 13.47 |
| 12 | 4.00 | 7.36 | 4.00 | 35.85 |
| 13 | 4.00 | 4.00 | 2.32 | 36.23 |
| 14 | 4.00 | 4.00 | 5.68 | 30.42 |
| 15 | 4.00 | 4.00 | 4.00 | 39.49 |
| 16 | 4.00 | 4.00 | 4.00 | 40.84 |

**(B) Cr removal efficiency**

| Run no. | PN3 biosurfactant (g/L) | NK8 biosurfactant (g/L) | pH | Removal efficiency of Cr (%) |
| --- | --- | --- | --- | --- |
| 1 | 2.00 | 2.00 | 3.00 | 3.55 |
| 2 | 2.00 | 2.00 | 5.00 | 7.21 |
| 3 | 2.00 | 6.00 | 3.00 | 2.61 |
| 4 | 2.00 | 6.00 | 5.00 | 6.74 |
| 5 | 6.00 | 2.00 | 3.00 | 23.41 |
| 6 | 6.00 | 2.00 | 5.00 | 25.95 |
| 7 | 6.00 | 6.00 | 3.00 | 35.75 |
| 8 | 6.00 | 6.00 | 5.00 | 37.92 |
| 9 | 0.64 | 4.00 | 4.00 | 2.06 |
| 10 | 7.36 | 4.00 | 4.00 | 32.67 |
| 11 | 4.00 | 0.64 | 4.00 | 10.26 |
| 12 | 4.00 | 7.36 | 4.00 | 12.14 |
| 13 | 4.00 | 4.00 | 2.32 | 15.27 |
| 14 | 4.00 | 4.00 | 5.68 | 19.30 |
| 15 | 4.00 | 4.00 | 4.00 | 33.33 |
| 16 | 4.00 | 4.00 | 4.00 | 33.51 |

1. **Formulations for Cu removal**


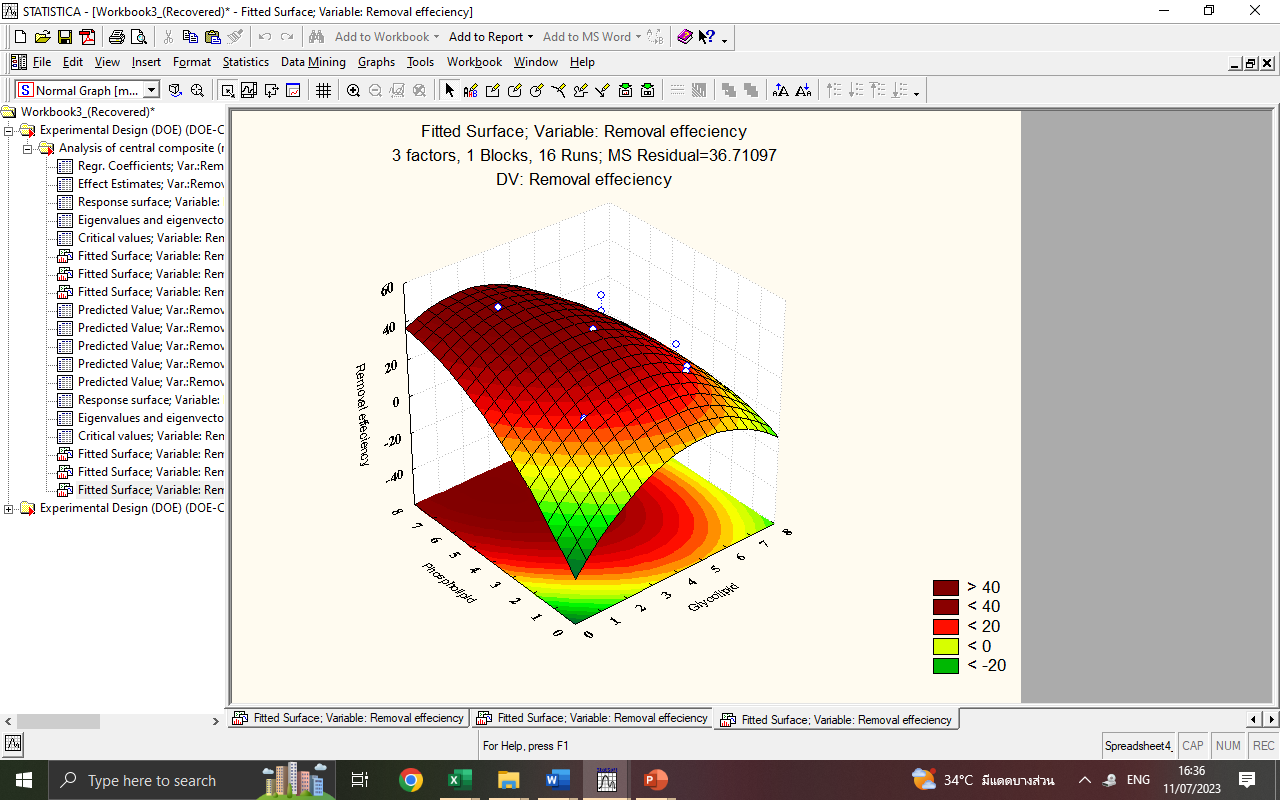


**Removal efficiency (%)**

**NK8 biosurfactant (g/L)**

**PN3 biosurfactant (g/L)**


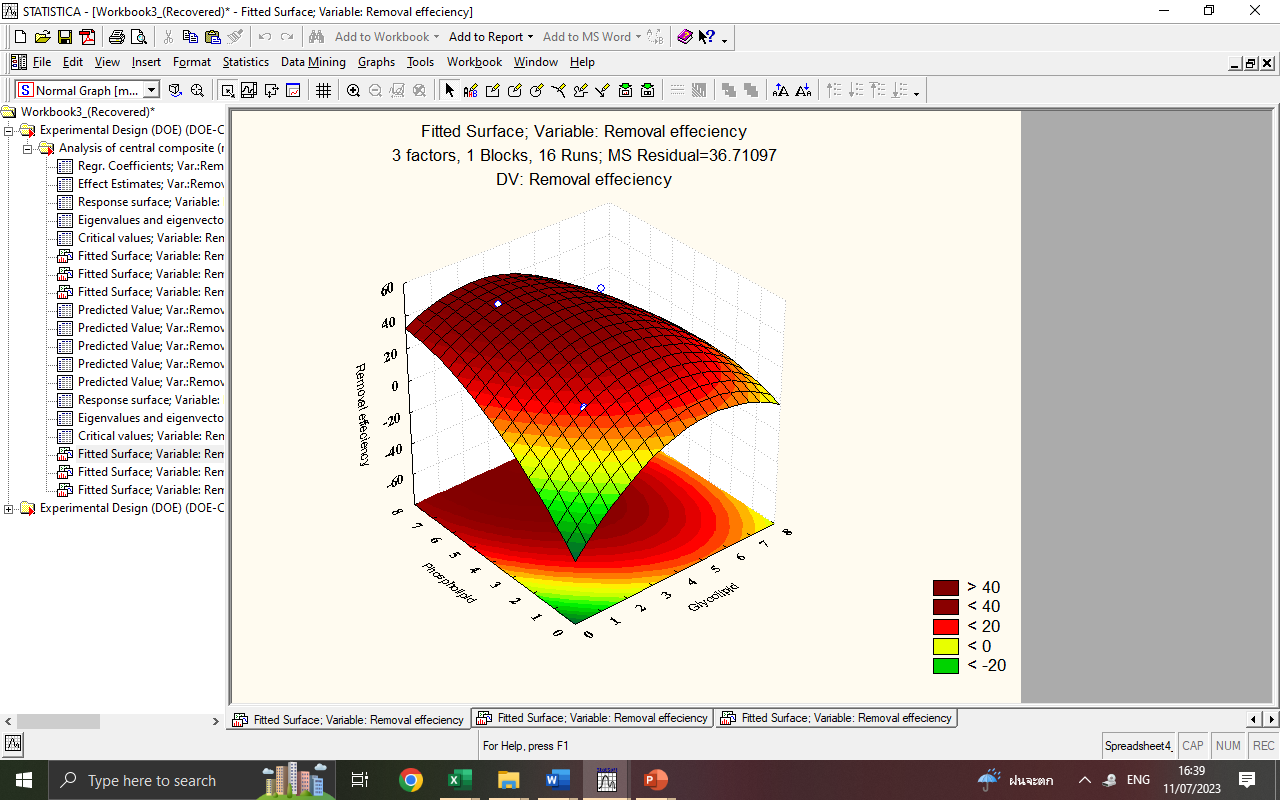


**B. Formulations for Cr removal**


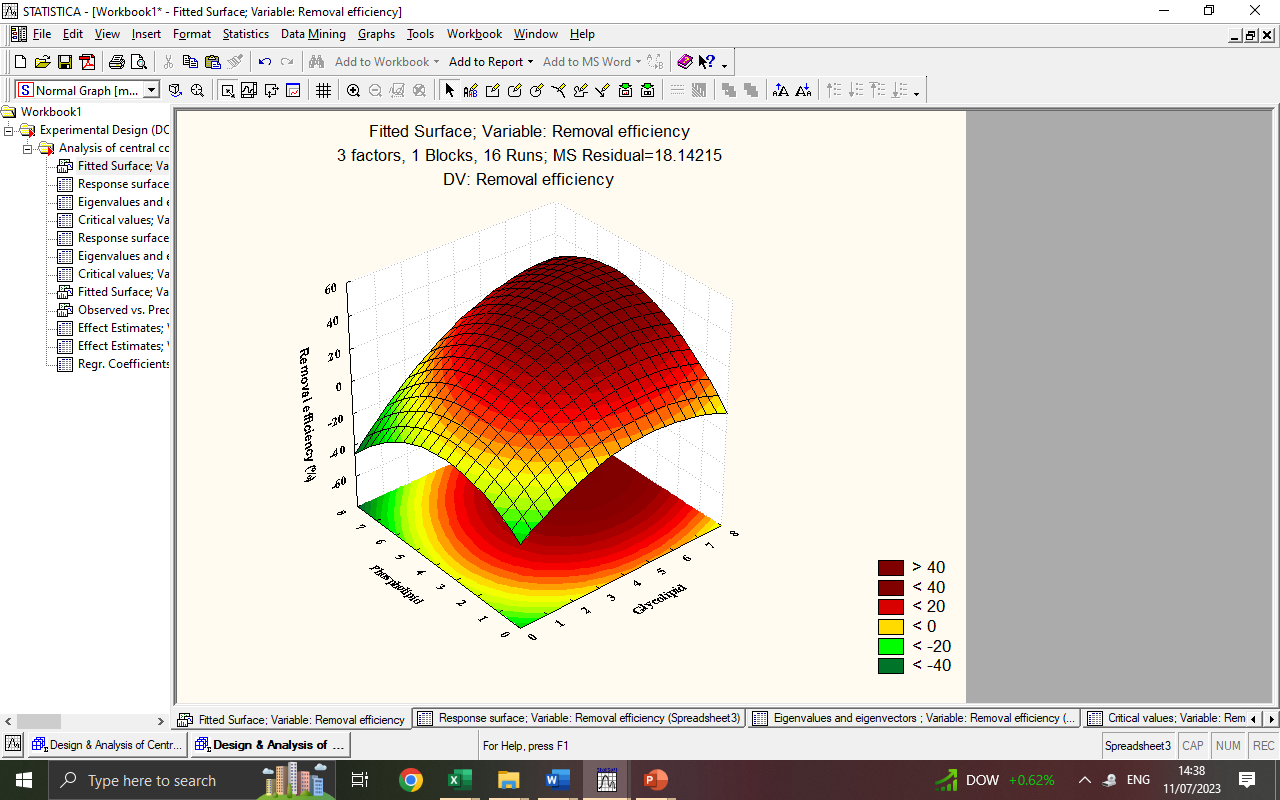


**Removal efficiency (%)**


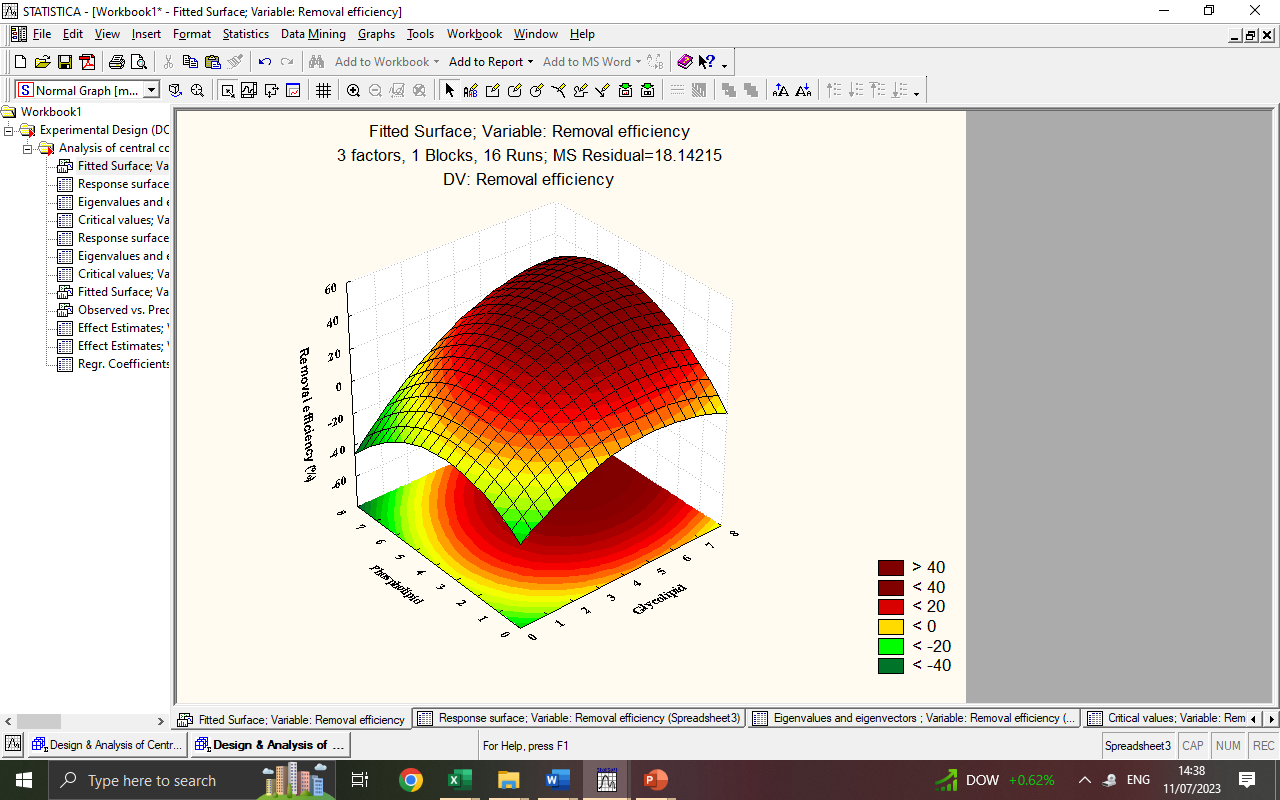


**NK8 biosurfactant (g/L)**

**PN3 biosurfactant (g/L)**

**Supplementary Figure 3.** Response surface plots for optimizing the compositions in biosurfactant-based washing agents at pH 4.0 for Cu (A) and Cr (B) removal from the industrial sludge.

**(C)**

**(D)**

**(A)**

**(B)**

**Supplementary Figure 4.** Mass of copper (A, B) and chromium (C, D) in the industrial sludge, washing agents, and rinsing water after repeated washing cycles. The industrial sludge was washed with formulation F3 (A, C) and formulation F4 (B, D).

**Supplementary Table 2.** Concentrations of Cu and Cr in the industrial sludge, washing agent, and rinsing water after repeated washing cycles. Two biosurfactant-based washing agent formulations (F3 and F4) were compared.

| Heavy metal | Formulation | Cycle | Sludge  (mg/kg) | Washing agent (mg/L) | Rinsing water (mg/L) |
| --- | --- | --- | --- | --- | --- |
| Cu | F3 | 1 | 2920.33±68.88 | 275.00±6.25 | 78.33±3.79 |
|  |  | 2 | 2607.67±67.84 | 131.67±7.51 | 70.00±2.00 |
|  |  | 3 | 2516.67±36.12 | 78.67±1.52 | 64.00±1.00 |
|  | F4 | 1 | 2768.00±147.50 | 283.00±4.58 | 144.00+2.00 |
|  |  | 2 | 2100.33±95.55 | 150.33±5.86 | 136.00±3.00 |
|  |  | 3 | 2075.00±15.39 | 84.00±3.00 | 71.67±2.89 |
| Cr | F3 | 1 | 3174.00±133.65 | 143.67±10.21 | 81.33±1.15 |
|  |  | 2 | 2934.00±41.33 | 127.67±2.08 | 61.67±17.92 |
|  |  | 3 | 2801.67±51.01 | 107.33±3.06 | 61.65±0.13 |
|  | F4 | 1 | 3489.33±276.80 | 112.67±9.50 | 52.41±0.23 |
|  |  | 2 | 3309±69.00 | 124.33±3.21 | 51.69±0.21 |
|  |  | 3 | 3155.67±22.19 | 104.67±2.08 | 51.39±0.06 |

**Supplementary Table 3.** PN3 biosurfactant identification by LC–ESI–QTOF–MS/MS in the positive and negative ionization modes.

| Retention time (min) | Mass peak (*m/z*) | Adduct | MS/MS | Tentative identification | Empirical formula | Error (ppm) |
| --- | --- | --- | --- | --- | --- | --- |
| 13.431 | 245.1398 | [M+H]+ | 171.1027,125.0967, 97.1018, 55.0546 | 3-hydroxydodec-6-enedioic acid | C_12_H_20_O_5_ | -5.91 |
| 15.463 | 264.2327 | [M+H]+ | 219.1759, 203.1440 | 2-{Bis[2-(3-aminopropoxy)ethyl]amino}ethan-1-ol | C_12_H_29_N_3_O_3_ | -17.15 |
| 16.421 | 171.1506 | [M+H]+ | 89.0713, 72.0445,55.0544 | 5-butyl-2-ethoxy-4,5-dihydro-1H-imidazole | C_9_H_18_N_2_O | -3.57 |
| 17.453 | 274.2767 | [M+H]+ | 106.0868, 88.0758, 70.0654, 57.0701 | C16 Sphinganine | C_16_H_35_NO_2_ | -9.64 |
| 23.0053 | 332.3353 | [M+H]+ | 240.2700, 91.0546, 58.0652 | N-Hexadecylbenzylamine | C_23_H_41_N | -12.41 |
| 35.816 | 338.3457 | [M-H]+ | 321.3175,303.3062 | 13-Docosenamide | C_22_H_43_NO | 35.816 |
| 11.785 | 247.1192 | [M-H]- | 173.0827,171.1032,111.0826,75.0096 | (e)-2-Methyl-2-buten-1-ol O-b-D-glucopyranoside | C_11_H_20_O_6_ | -1.98 |
| 12.701 | 173.0822 | [M-H]- | 111.0826,83.0511,67.0553,57.0357 | Dimethyl (S)-(+)-2-Methylglutarate | C_8_H_14_O_4_ | -1.55 |
| 13.405 | 261.1349 | [M-H]- | 187.0984,125.0980,97.0668,57.0352 | 2-oxo-5,8,12-trihydroxy-dodecanoic acid | C_12_H_22_O_6_ | -2.06 |
| 14.625 | 187.098 | [M-H]- | 125.0979,97.0668,80.0274,57.0354 | 3-Methylsuberic acid | C_9_H_16_O_4_ | -2.23 |
| 15.624 | 145.0872 | [M-H]- | 99.0822 | 7-Hydroxyheptanoic acid | C_7_H_14_O_3_ | -1.26 |
| 16.492 | 241.1088 | [M-H]- | 197.1197 | 3,3'-(2-Oxo-1,1-cyclohexanediyl) dipropanoic acid | C_12_H_18_O_5_ | -2.71 |
| 18.616 | 365.2109 | [M+Cl]- | 329.2336,211.1358,171.1003,139.1134 | (11E)-9,10,13-Trihydroxyoctadec-11-enoic acid | C_18_H_34_O_5_ | -2.39 |
| 20.6 | 169.0875 | [M-H]- | 125.0979,71.0511,55.0194 | 4-oxo-2E-nonenoic acid | C_9_H_14_O_3_ | -2.85 |

**Supplementary Figure 5.** Total ion chromatogram (TIC) and MS spectra of the purified PN3 biosurfactant in the positive (a) and negative (b) ionization modes.


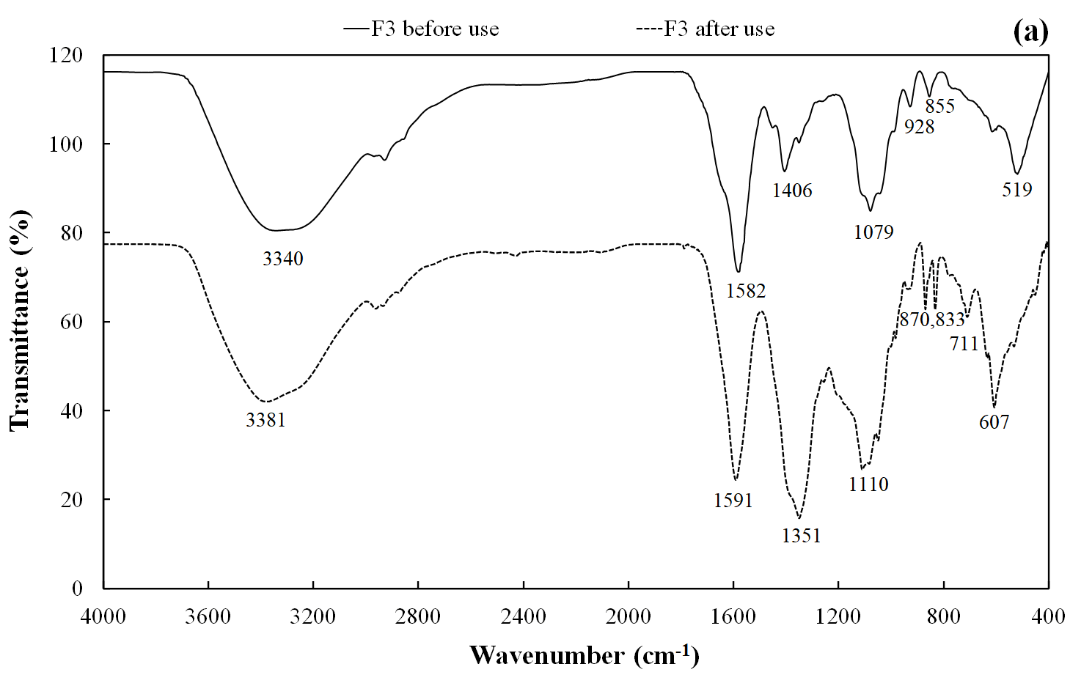


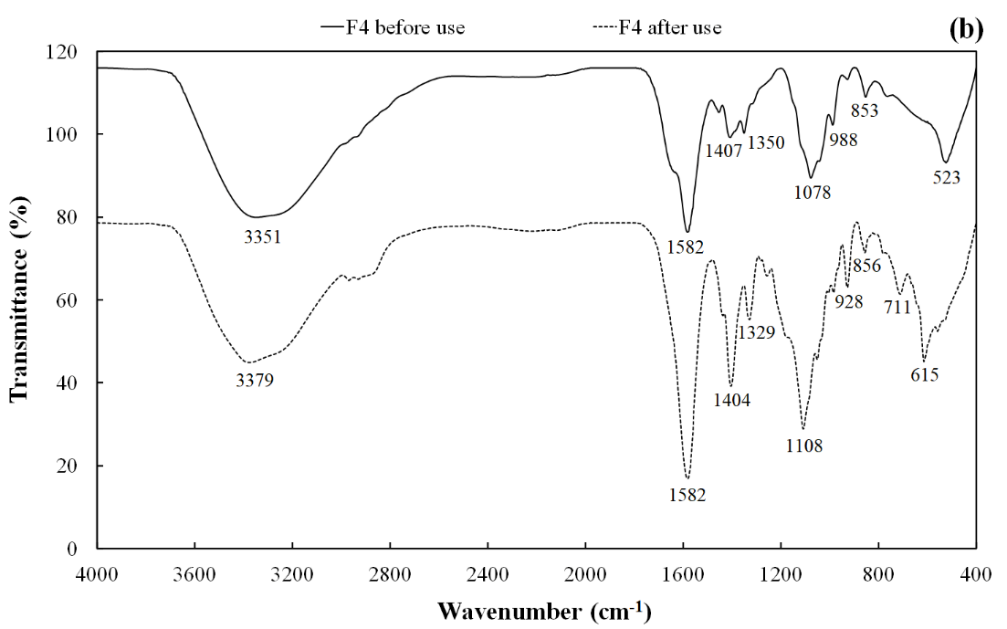


**Supplementary Figure 6.** FTIR spectra of biosurfactant-based washing agents F3 (a) and F4 (b) before and after use.

**(A)**

**(B)**

**Supplementary Figure 7.** Normalized XANES spectra of used Fe_3_O_4_ nanoparticles at Cr *K*-edge (A) and Cu *K*-edge (B). Effects of Fe_3_O_4_ nanoparticles concentrations, presence of biosurfactants, and pH on Cu and Cr were investigated with synthetic solutions containing 250 mg/L Cu(II), 150 mg/L Cr(VI), and 1 M EDTA without or with biosurfactants (+GP). The concentrations of Fe_3_O_4_ nanoparticles were described in the graphs as 50, 75, and 100 g/L. All experiments in Figure 7A tested at pH 4, while Figure 7B used pH 4.0 5.5. and 8.0. The reference spectra of standard Cr solutions (Cr_2_O_3_, CrCl_3_, CrO_3_, and stock Cr^6+^), Cu solutions (CuO and Cu_2_O), and Cu foil were shown for comparison.
